# Metachronous Bilateral Testicular Leydig-Like Tumors Leading to the Diagnosis of Congenital Adrenal Hyperplasia (Adrenogenital Syndrome)

**DOI:** 10.1155/2015/459318

**Published:** 2015-08-16

**Authors:** Josip Vukina, David D. Chism, Julie L. Sharpless, Mathew C. Raynor, Matthew I. Milowsky, William K. Funkhouser

**Affiliations:** ^1^Department of Urology, University of North Carolina, Chapel Hill, NC 27599, USA; ^2^Division of Hematology and Oncology, Department of Medicine, University of North Carolina, Chapel Hill, NC 27599, USA; ^3^Lineberger Comprehensive Cancer Center, Chapel Hill, NC 27599, USA; ^4^Division of Endocrinology and Metabolism, Department of Medicine, University of North Carolina, Chapel Hill, NC 27599, USA; ^5^Department of Pathology and Laboratory Medicine, University of North Carolina, Chapel Hill, NC 27599, USA

## Abstract

A 33-year-old male with a history of left testis Leydig cell tumor (LCT), 3-month status after left radical orchiectomy, presented with a rapidly enlarging (0.6 cm to 3.7 cm) right testicular mass. He underwent a right radical orchiectomy, sections interpreted as showing a similar Leydig cell-like oncocytic proliferation, with a differential diagnosis including metachronous bilateral LCT and metachronous bilateral testicular tumors associated with congenital adrenal hyperplasia (a.k.a. “testicular adrenal rest tumors” (TARTs) and “testicular tumors of the adrenogenital syndrome” (TTAGS)). Additional workup demonstrated a markedly elevated serum adrenocorticotropic hormone (ACTH) and elevated adrenal precursor steroid levels. He was diagnosed with congenital adrenal hyperplasia, 3*β*-hydroxysteroid dehydrogenase deficiency (3BHSD) type, and started on treatment. Metachronous bilateral testicular masses in adults should prompt consideration of adult presentation of CAH. Since all untreated CAH patients are expected to have elevated serum ACTH, formal exclusion of CAH prior to surgical resection of a testicular Leydig-like proliferation could be accomplished by screening for elevated serum ACTH.

## 1. Background

Testicular tumors in children and adults may be due to neoplasms or reactive hyperplasias. This paper considers the differential diagnosis of metachronous bilateral Leydig-like testicular tumors in an adult.

Primary testicular neoplasms are diverse, broadly categorized into germ cell tumors (seminoma, yolk sac tumor, embryonal carcinoma, choriocarcinoma, and teratoma) and sex cord/stromal tumors (Leydig cell tumors (LCT), Sertoli cell tumor, granulosa cell tumor, fibroma/thecoma, and undifferentiated tumors) [1]. Sex cord/stromal tumors are less common than germ cell tumors in children and adults [2]. LCTs represent 1–3% of all primary testicular tumors [3, 4] and up to 8% of pediatric primary testicular tumors [5]. LCTs in children and adults present as unilateral, unifocal testicular parenchymal masses in over 95% of cases [3]. Most are discrete solid tumors that displace adjacent parenchyma [3]. Microscopically, LCTs usually grow as sheets or septated lobules of minimally pleomorphic low N : C ratio cells with eosinophilic cytoplasm, cytologically bland nuclei, and prominent nucleoli [3–5]. Zona fasciculata-type vacuolated cells can be admixed or can predominate [3]. Spindle cell cytology, adipose metaplasia, and osseous metaplasia have been described [6]. Current immunohistochemical summary data predict that 85–95% of LCTs will show immunoreactivity for inhibin, Mart-1, and calretinin [7]. Two small series have shown that <10% of LCTs will show strong immunoreactivity for synaptophysin [8, 9].

Congenital adrenal hyperplasia (CAH) is a set of autosomal recessive genetic disorders characterized by loss-of-function defects in enzymes involved in the production of cortisol, aldosterone, and sex hormones [10, 11]. Roughly 90% of CAH cases are associated with loss-of-function mutations or translocations involving the* CYP21* gene that encodes the 21*α*-hydroxylase enzyme, and 5–8% are associated with loss-of-function mutations in the* CYP11B1* gene that encodes the 11*β*-hydroxylase enzyme [10, 12]. Less common yet are loss-of-function mutations in the* CYP17A1* gene that encodes the 17*α*-hydroxylase enzyme and loss-of-function mutations in the* HSD3β*2 gene that encodes the 3*β*-hydroxysteroid dehydrogenase enzyme. Loss of function of these enzymes leads to decreased production of cortisol and other steroid hormones. Cortisol deficiency leads to increased hypothalamic secretion of corticotropin-releasing hormone (CRH) and increased anterior pituitary secretion of adrenocorticotropic hormone (ACTH).

Increased serum ACTH in undertreated CAH causes hyperplasia of normal adrenal cortex bilaterally [10], hyperplasia of ectopic adrenal rests [13], and hyperplasia of “steroid” cells occurring normally in the testicular hilum [4, 14]. ACTH-driven hyperplasia of the testicular hilar “steroid” cells usually presents as nodules of cytologically bland oncocytic cells similar to normal Leydig cells [4, 15] although subsets of cells with clear, vacuolated cytoplasm similar to z. fasciculata may be seen [15]. Like LCTs, these hyperplastic steroid cell nodules are expected to show immunoreactivity for inhibin, Mart-1, and calretinin; unlike LCTs, they are also expected to show immunoreactivity for synaptophysin [8, 9].

Testicular hilar steroid cell hyperplastic nodules occur in up to 94% of CAH patients [15, 16]. Treatment guidelines now recommend screening testicular ultrasounds because the growth of these tumors can lead to testicular failure that is reversible with medical treatment [17]. Traditionally, these testicular steroid cell hyperplastic nodules have been detected as synchronous bilateral testicular hilar tumors [15, 16]. However, current ultrasonographic screening approaches frequently detect these nodules as unilateral testicular tumors in preteens that can develop into metachronous bilateral disease over time [18].

Different terms have been used to describe these testicular steroid cell hyperplasias, including “testicular tumors of the adrenogenital syndrome (TTAGS)” [4, 5], “testicular adrenogenital tumors” [19], “testicular tumors in congenital adrenal hyperplasia” [19], “testicular adrenal rest tumors (TART)” [10], “bilateral nodular hyperplasia of testicular adrenal rests” [10], and “adrenogenital syndrome-associated tumors” [20]. There is a distinction made in the urologic pathology literature between adrenal rests and the testicular hilar steroid cell hyperplasias [4]. Because these testicular hilar steroid cell hyperplasias respond to ACTH similarly to adrenal cortex and extratesticular adrenal rests and because the term is commonly used in the endocrinology, radiology, and urology literatures, we elect to refer to these tumors hereafter as “testicular adrenal rest tumors (TARTs).”

Comparison and contrast of the clinical, gross, micro, IHC, and endocrine features of LCTs and TARTs are summarized in Table 1. LCTs and TARTs are expected to share a similar Leydig-like cytology and to share a core immunophenotype of immunoreactivity for inhibin, Mart-1, and calretinin. In contrast, LCTs and TARTs frequently differ in terms of tumor uni/bilaterality at presentation, tumor uni/multifocality, tumor location in the testis, Reinke crystal prevalence, lipofuscin prevalence, serum ACTH, serum testosterone, tumor responsiveness to ACTH, and synaptophysin immunoreactivity.

Because TARTs frequently mimic Leydig cells cytologically, a large unifocal unilateral TART that displaces testicular parenchyma could easily be confused with an LCT in an adult in whom CAH is unrecognized. This diagnostic puzzle is less likely in a child, because precocious puberty would usually prompt an endocrine workup that would distinguish LCT from TART. This paper describes a case in which an adult presented with metachronous bilateral Leydig cell-like testicular tumors, prompting a workup for congenital adrenal hyperplasia (CAH), and was ultimately found to have CAH due to 3*β*-hydroxysteroid dehydrogenase deficiency.

## 2. Case Presentation

A 33-year-old male presented with a 2-month history of left testicular pain and swelling. He reported a history of hypospadias repair and bilateral cryptorchidism status after orchiopexy as a child. A scrotal ultrasound (US) revealed a heterogeneous 2.5 cm left testis mass and an incidental markedly heterogeneous 0.6 cm right testis nodule. A computed tomography scan of the chest and abdomen revealed no significant lymphadenopathy. He underwent left radical orchiectomy at an outside hospital, demonstrating a unifocal 3.5 × 3.0 × 2.2 cm LCT, without extratesticular invasion or vascular invasion. Nuclear atypia was seen, but no mitotic activity, coagulative tumor cell necrosis, vascular invasion, or Reinke crystals were noted. The Leydig-like proliferation was interpreted as a Leydig cell tumor, likely benign, staged as pT1.

**Figure 1:**
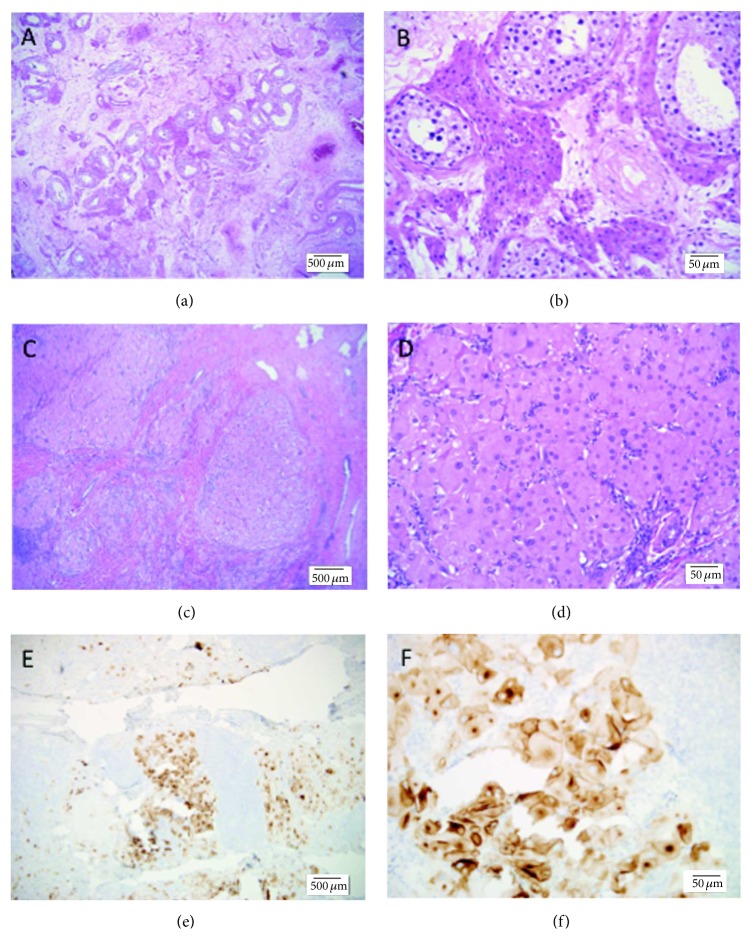
Morphology and IHC of the right testicular hilar tumor. (a) Uninvolved testis, H&E, 40x. (b) Uninvolved testis, H&E, 200x. (c) Testicular hilar tumor, H&E, 40x. (d) Testicular hilar tumor, H&E, 200x. (e) Testicular hilar tumor, synaptophysin, 40x. (f) Testicular hilar tumor, synaptophysin, 200x.

Two weeks after orchiectomy, he was referred to this institution for evaluation of a right testicular mass. He reported an unintentional 11-pound weight loss, night sweats, decreased libido, and erectile dysfunction. A repeat ultrasound of the right testis approximately 3.5 months after initial ultrasound revealed an increase in the size of the right testis nodule from 0.6 cm to 3.7 cm. No palpable mass was appreciated on initial or follow-up physical exams. The working clinical diagnosis at this juncture was a metachronous primary testicular malignancy. A metastatic workup was negative, and he underwent a right radical inguinal orchiectomy. Gross examination of the right testicular mass revealed a testicular hilar mass. Tissue sections showed nodules of oncocytic cells with low N : C ratios, prominent nucleoli, and focal lipofuscin, without Reinke crystals and without mitotic activity or coagulative necrosis (Figure 1). Immunostains showed reactivity for inhibin and synaptophysin. The pathologic diagnosis was “oncocytic proliferation, Leydig cell tumor versus testicular tumor of the adrenogenital syndrome,” that is, metachronous bilateral LCT versus TARTs.

He was referred to endocrinology for concern for congenital adrenal hyperplasia, and additional history revealed facial hair as an adolescent, an early growth spurt, frequent asthma flairs that responded to steroid dosing, and salt craving without a history of hypertension. In retrospect, he suffered nausea and vomiting after both surgical procedures. The first episode was associated with fever and resolved after administration of antibiotics. The episode after the second surgery resolved after changing to a different pain medication. He also reported a grandfather with a similar early puberty. After his first orchiectomy, he attempted sperm banking but was azoospermic. More directed physical exam revealed tan skin (although he was of Hispanic descent), pale striae on the abdomen, mild acanthosis nigricans of the neck, and multiple hyperpigmented chicken pox scars. Further testing demonstrated normal sodium levels, although there were no postoperative measurements, cortisol of 0.3 mcg/dL (normal range 4.5–22.7 mcg/dL), ACTH of 3050 pg/mL (normal range 10–60 pg/mL), and DHEA-S of 145 mcg/dL (normal range 65–334 mcg/dL). He had an elevated 17-OH pregnenolone to 17-OH progesterone ratio, consistent with 3*β*-hydroxysteroid dehydrogenase deficiency (3BHSD). He also had elevated renin activity supporting the salt-wasting variant of 3BHSD. His spontaneous puberty and late presentation point to a mild 3BHSD deficiency. He was referred to genetic testing but unfortunately was lost to follow-up. The findings of metachronous bilateral Leydig-like tumors associated with depressed cortisol and elevated serum ACTH are most consistent with bilateral TARTs in a patient with unrecognized CAH.

## 3. Discussion

The first TART case was reported in 1940 in a 3-year-old boy with marked salt cravings, an enlarged penis and scrotum, and pubic hair who died in the hospital just days after admission [21]. Laboratory testing revealed serum sodium of 111 mEq/L. Autopsy revealed hyperplasia of the androgenic cells of the adrenal cortex and bilateral masses in the testicles consisting of aberrant adrenal tissue.

TART prevalence as high as 94% is reported in patients with CAH [16, 22–24] and TART is reported to be the most important cause of male infertility in CAH patients [25]. Although the condition is benign, it can lead to infertility via two separate mechanisms. The first mechanism is physical compression and obstruction of the seminiferous tubules by the hyperplastic nodules [26, 27]. The second mechanism is impaired secretion of gonadotropins due to overproduction of adrenal androgens, leading to hypogonadotropic hypogonadism [16].

Accurate distinction between TART and LCT in adults without known CAH can be problematic. This is particularly true if the TART is unilateral or bilateral with a metachronous presentation (as in the current case). Even with foreknowledge of CAH in a patient with bilateral testicular masses, morphologic features may not allow distinction between TART and LCT [28]. However, clinical distinction between these diseases is of paramount importance, because the treatment differs (cortisol and/or mineralocorticoid replacement therapy for CAH, versus partial or complete orchiectomy for LCT). The two challenging clinical scenarios are (1) milder forms of CAH in adults, in which the testicular lesion may be the first presentation of CAH, and (2) optimally treated CAH, in which the tumors do not regress, thereby elevating LCT in the differential diagnosis [28, 29].

Clinical and radiographic features can be used to distinguish TART from LCT. TART presentation varies with age. In boys, the lesions are often unilateral [22] and the incidence of bilaterality increases with age [30]. In contrast, LCTs are usually unilateral, independent of age. Therefore, bilaterality strongly favors TARTs over LCTs. Radiographically, TARTs are primary in the testicular hilum, never primary in the parenchyma. In contrast, LCTs are primary in the testicular parenchyma, never primary in the hilum. Scrotal imaging via US or MRI can demonstrate the hilar/paratesticular TART nodules in CAH patients [18]. Computer tomography done for LCT staging in patients with untreated CAH may show bilateral diffuse enlargement of the adrenal glands, but a mass-like appearance can also occur [31] and an enhancement pattern has not been well established [32].

Endocrine features can be used to distinguish TART from LCT. TARTs represent reactive hyperplasias that develop in response to supraphysiologic ACTH, whereas LCTs are not ACTH-responsive and are not associated with supraphysiologic ACTH. Thus, preoperative serum ACTH can be used to screen for background CAH in patients with uni- or bilateral testicular tumors. Both LCTs and TARTs can be associated with alteration in serum testosterone. Most CAH is due to 21-hydroxylase deficiency, leading to high testosterone levels secreted by both the adrenals and the testes, until the TARTs get so big as to cause peritubular fibrosis leading to low testosterone. Thus, the serum testosterone in CAH is a function of the extent of testicular TART expansion [33, 34]. However, in our patient's rare form of CAH, 3BHSD type, his enzyme deficiency would limit production of testosterone. He had mildly elevated precursor steroids and slightly low testosterone level with elevated gonadotropins and azoospermia. This combination suggests two contributions to his gonadal dysfunction: first, partial enzyme deficiency as supported by his mild childhood course and late presentation and, secondly, testicular failure likely due to the TART. The elevated gonadotropins could also have further stimulated growth of the TART or differentiation toward LCT [35].

Gross and microscopic pathologic features can be used to distinguish TART from LCT. Bilaterality, multinodularity, dark brown color, hilar/paratesticular location, coarse fibrous stroma, and/or strong synaptophysin immunoreactivity favor TART over LCT [1, 8, 9]. Unifocality, yellow color, parenchymal location, scant/no fibrous stroma, and Reinke crystal formation favor LCT over TART. Leydig-like cytology, zona fasciculata-like cytology, adipose metaplasia, cytologic atypia, mitotic activity, lipofuscin pigment, and immunoreactivity for inhibin/Mart-1/calretinin are probably nonspecific findings [3, 4, 6, 8, 15]. Morphologic and immunophenotypic features of these Leydig-like proliferations overlap sufficiently that clinical and endocrinologic data become extremely valuable in achieving accurate pathologic diagnosis.

This patient's mild clinical symptoms of CAH likely contributed to the lack of clinical detection or treatment of his CAH, prolonging his exposure to high ACTH and further stimulating the ACTH-responsive steroid cells in the hilum of the residual testis. It has long been considered that growth of these lesions is due to chronic elevations of ACTH. TARTs have been shown to have ACTH receptors and to secrete steroids when stimulated with ACTH in vivo [36]. It can be speculated that the patient suffered worsening of his unrecognized adrenal insufficiency after his first surgery, which would have further raised his ACTH levels to the remarkably high levels documented when the diagnosis was finally considered. The rise in ACTH then stimulated growth of the second tumor after the first surgery. Workup leading to a CAH diagnosis will likely benefit this patient by assuring corticosteroid replacement during future stressful events such as surgical procedures. Interestingly, he was able to tolerate both anesthetics and procedures without issue, likely owing to their short duration and minimal morbidity. The postoperative nausea and vomiting could have been attributed to adrenal insufficiency; however other causes were plausible at the time.

## 4. Conclusions

We present a rare case in which pathology from a second orchiectomy led to a diagnosis of CAH due to (rare) 3*β*-HSD deficiency. This case highlights the challenges that urologists, radiologists, pathologists, and endocrinologists face when dealing with Leydig-like tumors in adults. We recommend a detailed history and physical examination as the first step in the evaluation of all patients with testicular masses, in addition to serum tumor markers and imaging. In this patient, the evolution of bilateral testicular masses over a short time interval could have prompted a more thorough discussion of past medical and family history, which would have revealed bilateral cryptorchidism, hypospadias, and a grandfather with early puberty. Taken together, this piece of information could have led to additional endocrine evaluation prior to surgical intervention. Because unilateral LCT and unilateral TART will share similar Leydig-like morphology and because both can involve the hilum and parenchyma, we recommend checking ACTH levels in all patients with a diagnosis of LCT with hilar involvement, as this may prompt formal preoperative workup for CAH.

## Figures and Tables

**Table 1:** Overlapping clinical presentations, morphologies, immunophenotypes, and associated endocrinopathies for LCTs and TARTs.

	Laterality	Gross appearance	Architecture	Cytology	Expected % cases with IHC reactivity	Expected serum ACTH	Expected serum testosterone
LCT	Unilateral	Intratesticular (parenchyma), solitary	Sheet-like or lobulated with fine fibrous bands	Leydig ± z. fascic. Reinke crystals in 30–40%	I 96% M 86% C 93% S < 10%	Normal	Increased

TART	Unilateral or Bilateral	Intratesticular (hilum), multifocal	Nodules with coarse fibrous bands	Leydig-like ± z. fascic. Reinke crystals absent	I (+) M (+) C (+) S > 88%	Increased	Increased/normal/low

z. fascic. = similar to adrenal zona fasciculata.

IHC = immunohistochemistry; I = inhibin; M = Mart-1; C = calretinin; S = synaptophysin.
